# Impact of low-frequency coding variants on human facial shape

**DOI:** 10.1038/s41598-020-80661-y

**Published:** 2021-01-12

**Authors:** Dongjing Liu, Nora Alhazmi, Harold Matthews, Myoung Keun Lee, Jiarui Li, Jacqueline T. Hecht, George L. Wehby, Lina M. Moreno, Carrie L. Heike, Jasmien Roosenboom, Eleanor Feingold, Mary L. Marazita, Peter Claes, Eric C. Liao, Seth M. Weinberg, John R. Shaffer

**Affiliations:** 1 Department of Human Genetics, Graduate School of Public Health, University of Pittsburgh, Pittsburgh, PA USA; 2 Department of Oral Biology, Harvard School of Dental Medicine, Boston, MA USA; 3 King Saud Bin Abdulaziz University for Health Sciences, Riyadh, Saudi Arabia; 4 Department of Human Genetics, KU Leuven, Leuven, Belgium; 5 Medical Imaging Research Center, UZ Gasthuisberg, Leuven, Belgium; 6 Center for Craniofacial and Dental Genetics, Department of Oral and Craniofacial Sciences, School of Dental Medicine, University of Pittsburgh, Pittsburgh, PA USA; 7 Department of Electrical Engineering, ESAT/PSI, KU Leuven, Leuven, Belgium; 8 Department of Pediatrics, University of Texas McGovern Medical Center, Houston, TX USA; 9 Department of Health Management and Policy, University of Iowa, Iowa City, IA USA; 10 Department of Orthodontics, University of Iowa, Iowa City, IA USA; 11 Department of Pediatrics, Seattle Children’s Craniofacial Center, University of Washington, Seattle, WA USA; 12 Department of Biostatistics, Graduate School of Public Health, University of Pittsburgh, Pittsburgh, PA USA; 13 Department of Surgery, Center for Regenerative Medicine, Massachusetts General Hospital, Shriners Hospital, Boston, MA USA

**Keywords:** Public health, Epidemiology

## Abstract

The contribution of low-frequency variants to the genetic architecture of normal-range facial traits is unknown. We studied the influence of low-frequency coding variants (MAF < 1%) in 8091 genes on multi-dimensional facial shape phenotypes in a European cohort of 2329 healthy individuals. Using three-dimensional images, we partitioned the full face into 31 hierarchically arranged segments to model facial morphology at multiple levels, and generated multi-dimensional phenotypes representing the shape variation within each segment. We used MultiSKAT, a multivariate kernel regression approach to scan the exome for face-associated low-frequency variants in a gene-based manner. After accounting for multiple tests, seven genes (*AR*, *CARS2*, *FTSJ1*, *HFE*, *LTB4R*, *TELO2*, *NECTIN1*) were significantly associated with shape variation of the cheek, chin, nose and mouth areas. These genes displayed a wide range of phenotypic effects, with some impacting the full face and others affecting localized regions. The missense variant rs142863092 in *NECTIN1* had a significant effect on chin morphology and was predicted bioinformatically to have a deleterious effect on protein function. Notably, *NECTIN1* is an established craniofacial gene that underlies a human syndrome that includes a mandibular phenotype. We further showed that *nectin1a* mutations can affect zebrafish craniofacial development, with the size and shape of the mandibular cartilage altered in mutant animals. Findings from this study expanded our understanding of the genetic basis of normal-range facial shape by highlighting the role of low-frequency coding variants in several novel genes.

## Introduction

Significant progress has been made in elucidating the genetic basis of human facial traits^1–3^. Genome-wide association studies (GWASs) have identified and replicated numerous common genetic variants associated with normal-range facial morphology^4–13^ (see implicated genes by facial regions in Fig S1); yet these variants cumulatively explain only a small fraction of the heritable phenotypic variation. Based on large-scale genomic studies of other complex morphological traits such as height^14–16^, we hypothesized that functional variants at hundreds or perhaps thousands of loci have yet to be discovered. While we expect that common variants, with a minor allele frequency (MAF) greater than 1%, account for most of the heritable variation in facial morphology, low frequency (MAF < 1%) genetic variants may also play an important role. An exome-wide study of human height, for example, discovered 29 low-frequency coding variants with large effects of up to 2 cm per allele^14^.

Our recent GWAS identified 17,612 common genetic variants associated with facial variation at 138 loci^13^. The success of this GWAS was attributed in part to an innovative data-driven phenotyping approach, in which the 3D facial surfaces were partitioned into hierarchically organized regions, each defined by multiple axes of shape variation. This approach allows for testing of genetic variants on facial morphology at multiple levels of scale—from the entire face (global) to highly localized facial regions (local). Extending this global-to-local analysis of facial traits to the analysis of low-frequency variants requires an appropriate and scalable statistical framework capable of accommodating the multivariate nature of the facial shape variables. A recently developed statistical approach, MultiSKAT^17^, was designed for this purpose and showed desirable performance in its original development.

In this study, we evaluated the influence of low frequency coding variants, captured by the Illumina HumanExome BeadChip, on normal-range facial morphology in 2,329 individuals. We applied multivariate gene-based association testing methods to multi-dimensional facial shape phenotypes derived from 3D facial images. The results of our analyses pointed to novel genes, including at least one involved in orofacial clefts and several others with no previously described role in craniofacial development or disorders. We provided experimental evidence of our genetic association results through expression screening and knockout experiments in a zebrafish model. These results enhance our understanding of the genetic architecture of human facial variation.

## Materials and methods

### Ethics statement

Institutional ethics (IRB) approval was obtained at each recruitment site (University of Pittsburgh Institutional Review Board #PRO09060553 and #RB0405013; UT Health Committee for the Protection of Human Subjects #HSC-DB-09-0508; Seattle Children’s Institutional Review Board #12107; University of Iowa Human Subjects Office/Institutional Review Board #200912764 and #200710721). All adult subjects gave their written informed consent prior to participation, and for children, written consent was obtained from a parent or legal guardian. All procedures performed in this study was conducted in accordance with the guidelines of the Declaration of Helsinki. All experimental protocols using zebrafish were approved by the Animal Care and Use Committees of Massachusetts General Hospital and carried out in accordance with institutional animal care protocols.

### Sample and phenotyping

**Figure 1:**
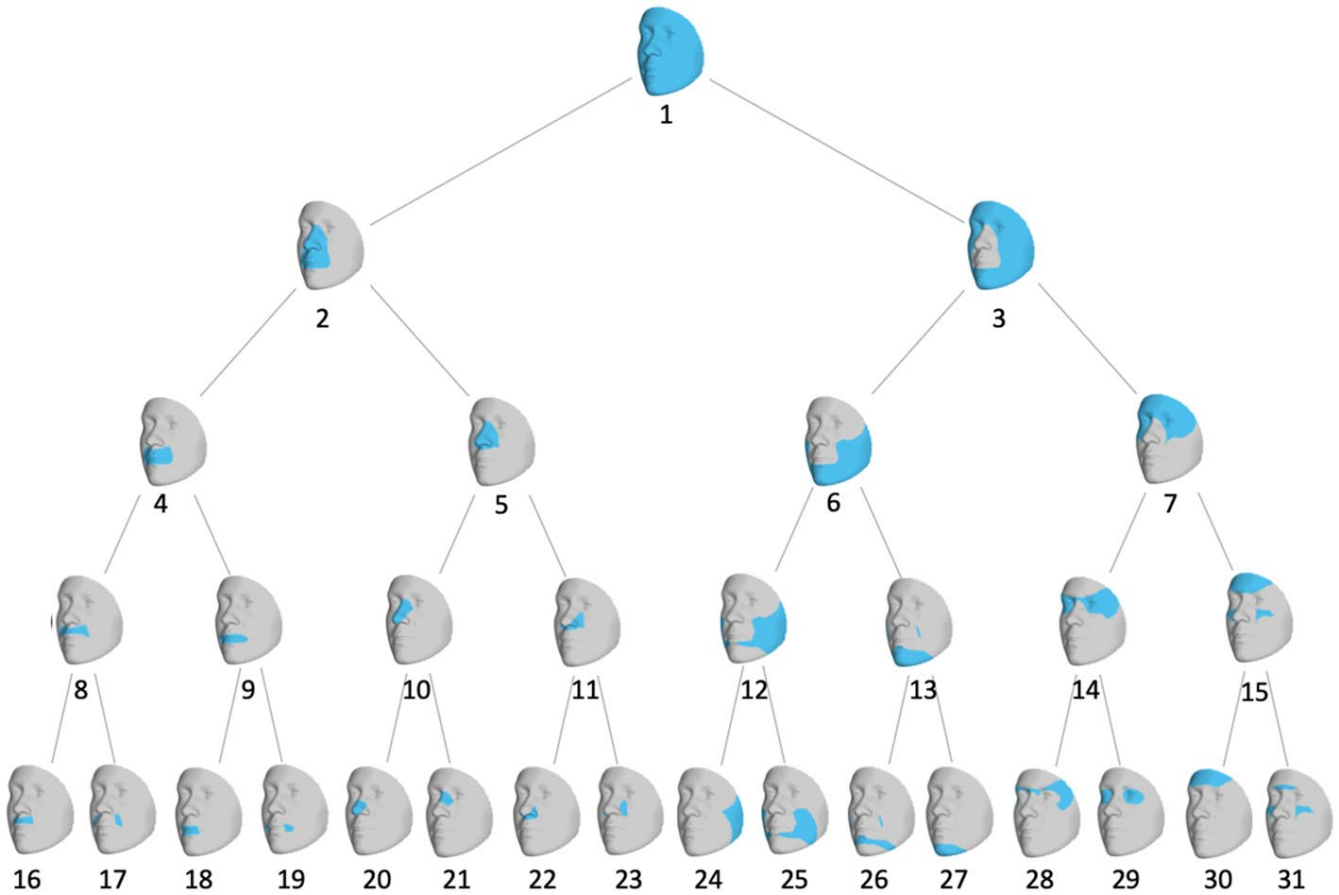
Hierarchical clustering of facial shape. Global-to-local facial segmentation obtained using hierarchical spectral clustering. Segments are colored in blue. The highest-level segment representing the full face was split into two sub-segments, and this bifurcation process was repeated until a five-level hierarchy comprising 31 segments was formed.

The study cohort comprised 2329 unrelated, healthy individuals of European ancestry aged 3–40 years. Participants were eligible if they had not experienced facial trauma, major surgery, congenital facial anomalies that could potentially affect their natural facial structure. 3D images of each participant’s resting face were captured via digital stereophotogrammetry using the 3dMD face camera system. The data-driven phenotyping approached has been described in detail in a previous work^5^. Briefly, approximately 10,000 points—“quasi-landmarks”—were automatically placed across the facial surface, by a non-rigid registration of a standard facial template onto each surface. The result is that each quasi-landmark represents the same facial position across all participants^18^. The configurations were then co-aligned to their mean using generalized Procrustes analysis (GPA). The quasi-landmarks were then clustered into groups of co-varying component points in order to partition the full face into two segments. GPA was repeated within each of the two segments, and the process was continued for a total of four iterations to generate a hierarchy of 31 facial segments (which we call modules) comprising overlapping groups of quasi-landmarks. The hierarchical structure is illustrated in Fig. 1, where modules formed successive levels representing the shift from more globally integrated to more locally focused morphology. Shape variation within each module was represented by the 3D coordinates of all quasi-landmarks contained therein. To reduce the dimensionality, principal components analysis and parallel analysis were performed on the quasi-landmarks. The result was a set of 31 multivariate phenotypes made up of 8–50 principal components (PCs) that jointly captured near complete shape variance. The effects of sex, age, height, weight, facial size and genetic ancestry were corrected for at the phenotyping stage, by regressing facial shape on these variables using partial least-squares regression. These facial module phenotypes were successfully used in our previous GWAS of common variants^5^, which demonstrated a clear advantage of this data-driven multivariate modeling approach for gene-mapping studies over the traditional utilization of a priori^8^ and univariate^7^ facial traits.

In addition to the phenotype quality control process described in^5^, we further examined the phenotypic distribution of each module for extreme outlier faces, as phenotypic outliers may adversely impact low-frequency variant tests^19^. To accomplish this, we looked at both the joint and the pairwise distribution of all PCs underlying each module. We visualized quantile–quantile (Q–Q) plots of chi-squared quantiles versus robust squared Mahalanobis distances to identify outliers that deviated from the rest of the sample. Mahalanobis distance is a metric measuring how far an observation is to the center of the joint distribution (centroid equivalent in a multivariate space). We identified one individual who was an outlier for several PCs in module 27 (chin), and revisited the associated facial images to confirm data validity and sample eligibility. This individual was excluded from any subsequent analysis involving module 27.

### Genotyping

Participants were genotyped by the Illumina OmniExpress + Exome v1.2 array, which included approximately 245,000 coding variants in the exome panel. Standard data cleaning and imputation procedures were implemented. Imputed genotypes with a certainty above 0.9 were used to fill in any sporadic missingness among genotype calls of the directly genotyped variants. We did not include any wholly unobserved, imputed SNPs in this analysis. Ancestry PCs based on common LD-pruned SNPs were constructed and regressed out from the multivariate traits to adjust for population structure.

### MultiSKAT

MultiSKAT^17^ was specifically developed for testing sets of variants, in this case coding variants within genes, for association with a multivariate trait. Testing low-frequency variants in aggregate can improve power compared to individual tests of each variant. The tool is flexible in relating multiple variants collectively to multiple phenotypes through the use of several choices of kernels and includes an omnibus test to obtain optimal association p-values by integrating results across different kernels via Copula. This capability of accommodating multivariate phenotypes fits well with our analysis of facial modules, as each module was composed of several independent PCs. MultiSKAT can be applied to both common and rare variants, although our analysis considered low-frequency variants exclusively.

MultiSKAT uses a phenotype kernel to model how one variant affects multiple traits, and a separate genotype kernel to specify how multiple variants influence one trait. In reality, these effects are often not known a priori, and the true relationship can be a mixture of different effects. We used the heterogeneous and homogeneous phenotype kernels, which are appropriate when the set of traits analyzed are orthogonal PCs. We used the Sequence Kernel Association Test (SKAT) and burden test as the genotype kernel and performed the omnibus test in MultiSKAT to aggregate results across the 2 × 2 kernel combinations.

### Gene-level analysis

Genome-wide coding variants with MAF < 1% were aggregated into genes. Per the developer’s suggested practice for using the MultiSKAT method, we filtered out variants with three or fewer minor alleles to ensure that there is no inflation in MultiSKAT test statistics. We excluded genes with less than two qualified variants, leading to 31,347 variants in 8091 genes being tested. When grouping multiple variants into a gene, MultiSKAT assigns larger weights to rarer variants. We applied a Bonferroni threshold to declare significance. To account for the correlation among partially overlapping facial modules, we used a procedure based on eigenvalues as proposed by Li and Ji^20^ and computed that the effective number of independent modules was 19. The threshold for significance was therefore set as p < 3.3 × 10^–7^ [i.e., 0.05/(8091 × 19)]. The phenotypic effects of identified genes on face were visualized by creating and comparing the average facial morphs in individuals who had variants in a certain gene and those who do not carry any variants.

Gene-set enrichment analysis was carried out using GREAT^21^, FUMA^22^ and ToppFun^23^. Expression of genes were looked up in the GTEx database^24^. Following our hypothesis that genes influencing typical facial presentation may also be involved in facial anomalies, we examined whether any genes identified by MultiSKAT were associated with non-syndromic cleft palate with or without cleft lip (NSCL/P) by retrieving association p-values from a past study of our group, where we performed a gene-based low-frequency variant association scan on NSCL/P^25^.

### Variant-level analysis

For genes highlighted by MultiSKAT, we scrutinized the quality of genotype calls by inspecting allele intensity cluster plots. We further performed association tests of individual SNPs using MultiPhen^26^. MultiPhen works by finding the linear combination of PCs that is mostly associated with the genotypes at each SNP and is robust when variants with low frequencies are tested against non-normal phenotypes. Variant level functional prediction was performed using CADD^27^. CADD is a comprehensive metric that weights and integrates diverse sources of annotation, by contrasting variants that survived natural selection with simulated mutations. The scaled CADD score expresses the deleteriousness rank in terms of order of magnitude. A score of 10, for instance, is interpreted as ranking in the top 10% in terms of the damaging degree amongst reference genome SNPs, and a score of 20 refers to 1%, 30 to 0.1%, etc. Variant identifiers and chromosomal locations are indicated according to the hg19 genome build. Individual variants were searched in literature and PhenoScanner^28^ for existing human phenotype associations.

We quantified the magnitude of phenotypic effect of individual low-frequency variants by the difference between averaged faces of variant carriers (those who were heterozygotes; there was no homozygotes for the low-frequency variants tested) and non-carries, which was further compared with the effects of significant common variants identified in the prior GWAS of the same multidimensional traits^5^. Specifically, the centroids of the multidimensional space defined by PCs in a certain module were computed separately for people carrying the variant and people who do not carry the variant. Then the Euclidean distance between the two centroids was calculated as a measure of variant effect size.

### Expression screen of candidate genes in zebrafish

The whole-mount RNA in situ hybridization (WISH) for *ar, cars2, ftsj1, hfe, ltb4r, telo2, nectin1a* and *nectin1b* was performed on wild type zebrafish embryos at 24 hpf and 48 hpf as described by Thisse et al.^29^. All wild type embryos were collected synchronously at the corresponding stages and fixed in 4% paraformaldehyde (PFA) overnight. T7 RNA polymerase promoter was added to the reverse primers and was synthesized with antisense DIG-labeled probe in order to generate antisense RNA probe. The probe primers for *ar* are: forward 5′-GTCCTACAAGAACGCCAACG-3′ and reverse 5′-GGTCACAGACTTGGAAAGGG-3′ at 59 °C. The *cars2* probe primers are: forward 5′-ATCTGGGTCATGCGTGTTCA-3′ and reverse 5′-GGATTCCTGTGGTGCTTGGT at 59 °C. The *ftsj1* probe primers are: forward 5′-GGCGAGAAGTGCCTTCAAAC-3′ and reverse 5′-AGTCGTGCTTGTGTCTGGTT-3′ and *hfe* probe primers are: forward 5′-GGGGATGGATGCTTCTACGA-3′ and reverse 5′-CGCGCACACAAAATCATCAC-3′ at 59 °C. The *ltb4r* probe primers are: forward 5′-GACGGTGCATTACCTGTGC-3′ and reverse 5′-AGTCTTGTCCGCCAAGGTC-3′ at 58 °C. The primers for *telo2* are: forward 5′-GCTCCACTGGTGAGAGTGAG-3′ and reverse 5′-GTCAGCTGAGGAGAGTCTGCG-3′. The primers for *nectin1a* probe are: forward 5′-AACACCCAGGAGATCAGCAA-3′ and reverse 5′-CCTCCACCTCAGATCCGTAC-3′ at 57 °C and the *nectin1b* probe primers are: forward 5′-TGCTAACCCAGCATTGGGAG-3′ and reverse 5′-GGTTCTTGGGCATTGGAGGA-3′ at 59 °C. Embryos were mounted using glycerol and imaged using Nikon AZ100 multizoom microscope.

### Phenotype of mutant zebrafish

Zebrafish adults and embryos were obtained and maintained as described by Kimmel et al.^30^. Zebrafish *nectin1a* mutants were generated by transgene insertion Tg(Nlacz-GTvirus) in Chr 21: 21,731,876–21,731,886 (Zv9), and obtained from Zebrafish International Resource Center, allele Ia021885Tg (ZIRC catalog ID: ZL6899.07). The retroviral-mediated insertional mutagenesis inserts a molecular tag in the DNA and isolates the allele of interest. Therefore, this will induce a frameshift and probably causing either nonsense-mediated mRNA decay or a truncated protein^31,32^. The PCR genotyping primers for *nectin1a* are: forward 5′-TTAGACCAGCCCACCTCA-3′ and reverse 5′-AATATGAAATAGCGCCGTTGTG-3′ at 62 °C.

Alcian blue staining was performed as described by Walker et al.^33^. The craniofacial cartilages were dissected and flat-mounted and then imaged using Nikon AZ100 multizoom microscope. After imaging, each embryo tail was placed in a PCR tube for genotyping. The protocol was used as described by^34^ with modification of using fresh embryos without fixation.

## Results

**Figure 2:**
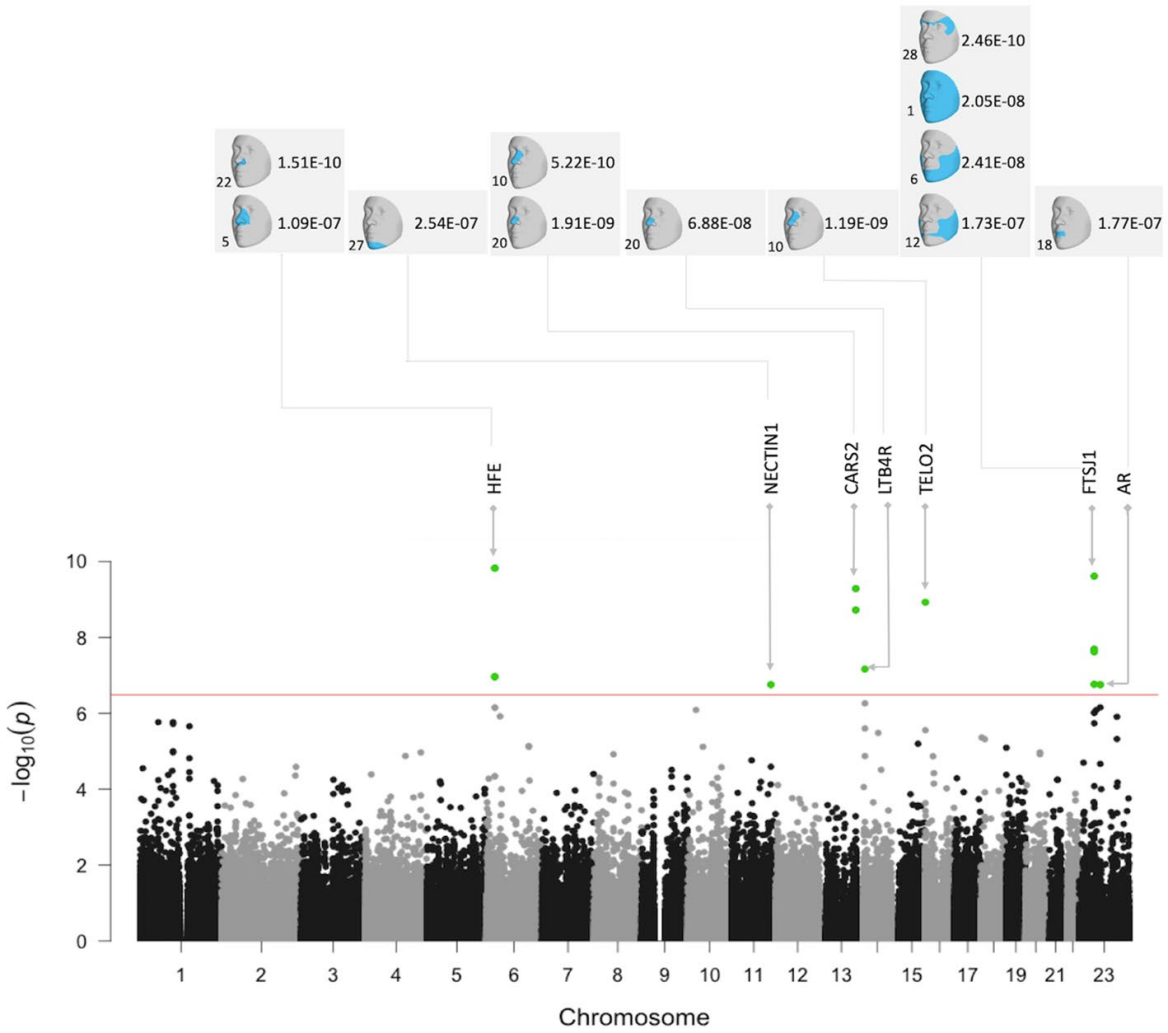
Composite Manhattan plot showing results across 31 facial modules. Manhattan plot showing the position of genes on the x axis and MultiSKAT p-values on the y axis. A total of 31 points are plotted for each gene, representing their p-values in each of the 31 modules. The red horizontal line indicates the significance threshold (3.3 × 10^–7^). The associated facial modules and the corresponding p-value for each gene that surpassed the threshold (marked as green dots) are shown above the Manhattan plot. The numbers to the bottom left of the facial images indicate the module identifiers in Fig. 1.

**Figure 3:**
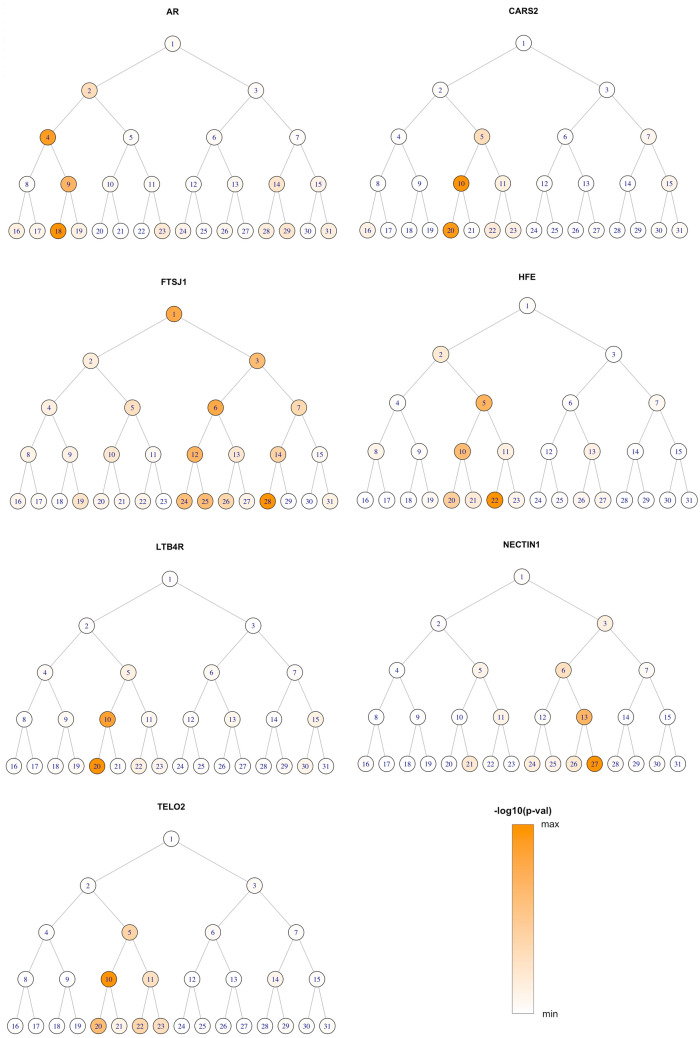
Module-wide association results for significant genes. For each gene, the –log10 p*-*value is shown as color shades ranging from min to max, for 31 facial segments arranged the same way as Fig. 1. The global-to-local phenotyping enabled the discovery of genetic effects at different scales.

In the gene-based test of exome-wide low-frequency variants, seven genes were significantly associated with one or more facial modules (*HFE, NECTIN1, CARS2, LTB4R, TELO2, AR,* and *FTSJ1;* Fig. 2 and Table 1). Three of them showed associations with more than one module. Figure 3 and Table S1 show the results of these genes in multiple modules. Figure 3 shows the association signals propagating along the branching paths from the more global segments to the more local segments. Four genes (*HFE, CARS2, LTB4R,* and *TELO2*) were associated with nose-related modules, and the others were associated with the shape of chin, mouth, and cheek. *FTSJ1* had broad associations in the full face as well as in local regions, while the effects of other genes were more confined to only local modules. We observed well-calibrated test statistics and little evidence of inflation as shown in the Q–Q plots (Fig S2). Genes which did not reach our significance threshold but had a small p-value are listed in Table S2.

**Table 1:** Single variant association and functional prediction for variants contributing to the gene-level significance.

Chr	Gene	Gene info	Gene-level association		Variant-level association							
			Module^a^	MultiSKAT P-value^a^	SNP	Pos (hg19)	Ref/Alt^b^	Function^c^	CADD score^d^	MAF (%)	Module^e^	MultiPhen P-value^e^
6	*HFE*	Homeostatic iron regulator, binds to transferrin receptor (TFR) and reduces its affinity for iron-loaded transferrin	5, **22**	1.51E-10	rs149342416	26,087,686	G/C	Arg6Ser	15.3	0.09	22	0.07
					rs143662783	26,087,718	C/G	Thr17Ile	13.4	0.09	5	0.87
11	*NECTIN1*	Nectin 1, cell adhesion molecule	27	2.54E-07	rs142863092	119,548,369	G/A	Arg210His	25.2	0.09	27	1.08E−03
					rs137991779	119,549,425	G/A	Gly44Ser	29.2	0.11	27	0.15
13	*CARS2*	Cysteinyl-tRNA synthetase 2, mitochondrial	**10**, 20	5.22E-10	rs151097801	111,296,817	C/T	Pro138Leu	22.4	0.09	20	0.12
					rs117788141	111,357,899	G/A	Val69Ile	28	0.09	10	0.01
14	*LTB4R*	Leukotriene B4 receptor 1, receptor for extracellular ATP > UTP and ADP	20	6.88E-08	rs143666989	24,780,865	A/G	Gln332Arg	16.6	0.11	20	0.11
					rs148153989	24,780,915	A/T	Met349Leu	12.5	0.09	20	0.59
16	*TELO2*	Telomere length regulation protein homolog, regulate DNA damage response	10	1.19E-09	rs140903666	1,544,313	G/A	Ala11Thr	6.3	0.22	10	8.21E−04
					rs144863771	1,544,314	C/A	Ala11Asp	10.7	0.22	10	8.21E−04
					rs147858841	1,555,541	C/T	Ala132Val	9.4	0.11	10	0.43
23	*AR*	Androgen receptor, steroid hormone receptors	18	1.77E-07	rs142280455	66,905,875	A/G	Ser598Gly	22.4	0.13	18	0.81
					rs137852591	66,941,751	C/G	Gln267Glu	25	0.13	18	3.91E−03
23	*FTSJ1*	Putative tRNA (cytidine(32)/guanosine(34)-2′-O)-methyltransferase	1, 6, 12, **28**	2.46E-10	rs142932029	48,341,118	G/A	Ser161Asn	7.4	0.08	28	1.59E−14
					rs201095751	48,341,414	C/T	Splice site	0.1	0.11	12	0.1

^a^For genes associated with multiple facial modules, the most significant module is in bold and only its p-value is shown.

^b^Alleles are listed as alternative/reference alleles on the forward strand of the reference genome.

^c^For missense variant, amino acid substitution is given.

^d^Bioinformatic prediction of variant effect, higher score indicates greater damaging effect.

^e^Variants were tested against all module(s) with gene-level significance, and for genes associated with multiple modules, only the module yielding the smallest p-value in the variant-level test is shown.

**Figure 4:**
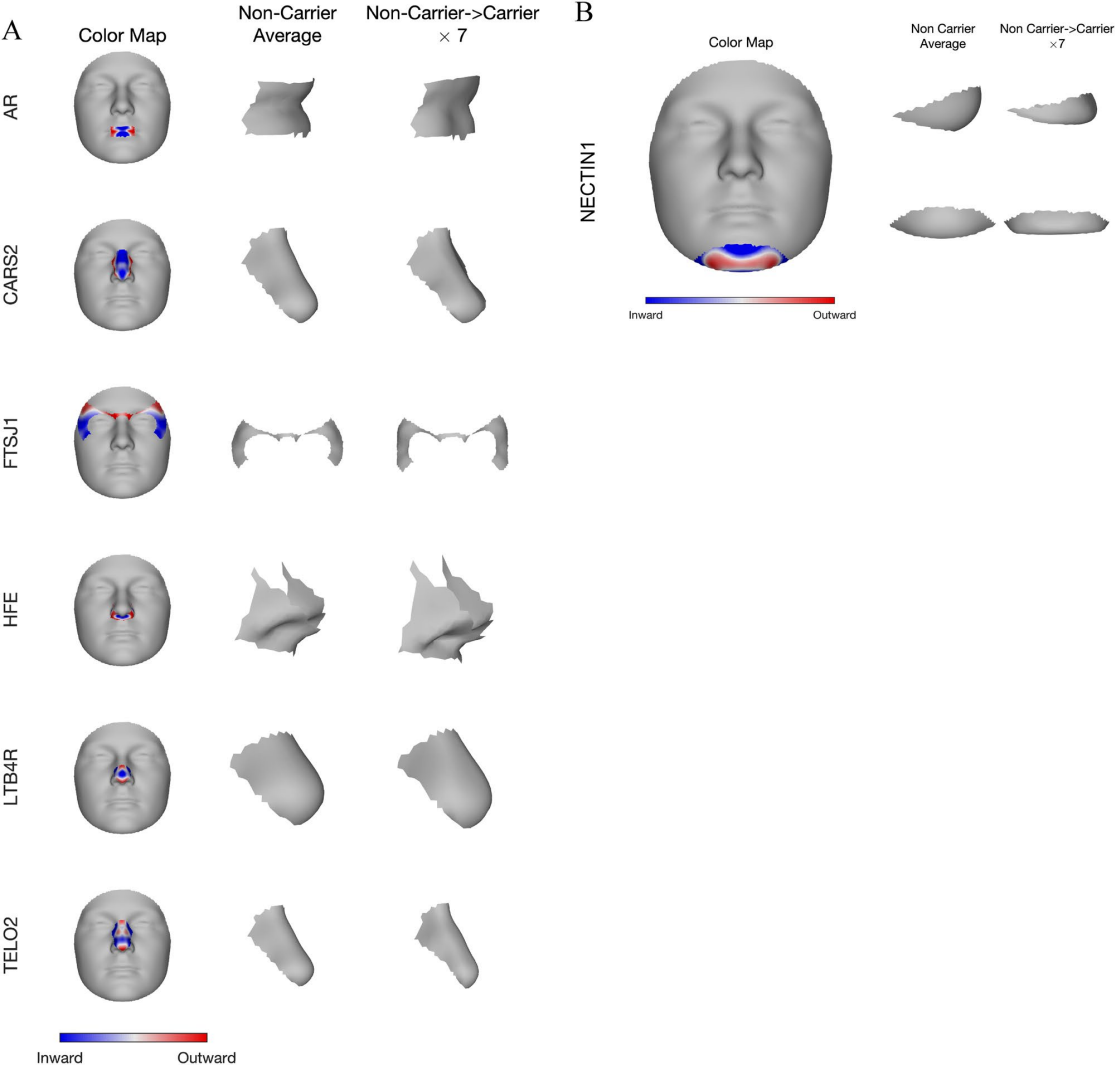
Phenotypic effect of the seven identified genes in their top associated module. Blue and red indicate a local shape depression and protrusion, respectively, due to carrying the low-frequency variants in the gene. (**A**) First column shows gene effect on a representative module placing on the full face; middle column shows the lateral view of the average shape of the corresponding module among people who do not carry any variant in the gene; right column shows the change in the shape of the same module, from non-carrier to carrier, multiplied by a constant (7), to make the changes more visibly distinctive. (**B**) For *NECTIN1* gene, we show both lateral (top) and frontal (bottom) view of its effect on chin shape. *NECTIN1* variant carriers on average displayed a sharper, more protruding chin.

To visualize the effects of these genes on facial shape, we created the average module shape in non-carriers of the low-frequency variants for each gene, and a corresponding morph showing the change in shape from non-carriers to carriers (Fig. 4). Blue and red indicate a local shape depression and protrusion, respectively, due to carrying any low-frequency variants. As an example, panel B in Fig. 4 shows that *NECTIN1* variants shape the chin into a sharper and more protruding structure.

We employed various bioinformatics tools to explore the functions associated with the set of identified genes. Enrichment was detected for a variety of biological processes (Fig S3), especially ion-, metabolism-, transport- and regulation-related processes. Enriched gene ontology (GO) molecular functions included signaling receptor and protein binding activity. Two genes with relatively well characterized functions (*HFE* and *AR)* contributed a lot to these enrichment results. In the GTEx database, these seven genes showed measurable expression level in adipose, skin and skeletal muscle tissue (Fig S4), among which the strongest expression was seen for *NECTIN1* in skin. However, these expression data should be interpreted with caution since GTEx tissues do not necessarily reflect the embryonic processes associated with craniofacial development.

To explore whether facial genes also affect the risk of orofacial clefts, results of gene-based associations of low-frequency (MAF < 1%) variants with NSCL/P were retrieved from Leslie et al. 2017. Two out of the seven were not available from that study. Table S3 shows the SKAT and CMC test results for the other five genes in the European, Asian, South American and the combined samples. Two associations passed a Bonferroni corrected threshold for 40 tests (5 genes × 4 populations × 2 type of tests)—*TELO2* with a CMC p-value = 6.5 × 10^–4^, and HFE with a CMC p-value = 1.1 × 10^–3^, both in the combined population of all ancestry groups.

Single variants in the genes showing significant associations in the gene-based tests were further tested individually with the corresponding facial modules (Table 1). Six SNPs showed nominal associations (p-value < 0.05) and the top association involved SNP rs142932029 in *FTSJ1* with module 28 (p-value = 1.59 × 10^–14^). As shown in Fig S5, these low-frequency variants had larger effects compared to previously reported common variants^5^.

Most of the individual variants appeared at frequencies much lower than 1%, and all encode nonsynonymous substitutions except one splice site SNP in *FTSJ1*. Variants in *NECTIN1, CARS2* and *AR* are predicted to be deleterious according to their CADD score (details in Table S4). SNP rs137991779 in *NECTIN1* has a CADD score of 29.2, interpreted as ranking in the top 0.12% in terms of deleteriousness among variants across the whole genome. PhenoScanner linked those variants with a variety of human traits/disorders in previous studies (Table S5, mostly from UK Biobank), including height, vascular diseases, osteoporosis, neoplasms etc., suggesting that coding variants influencing facial shape may be pleiotropic and play roles in other biological processes.

**Figure 5:**
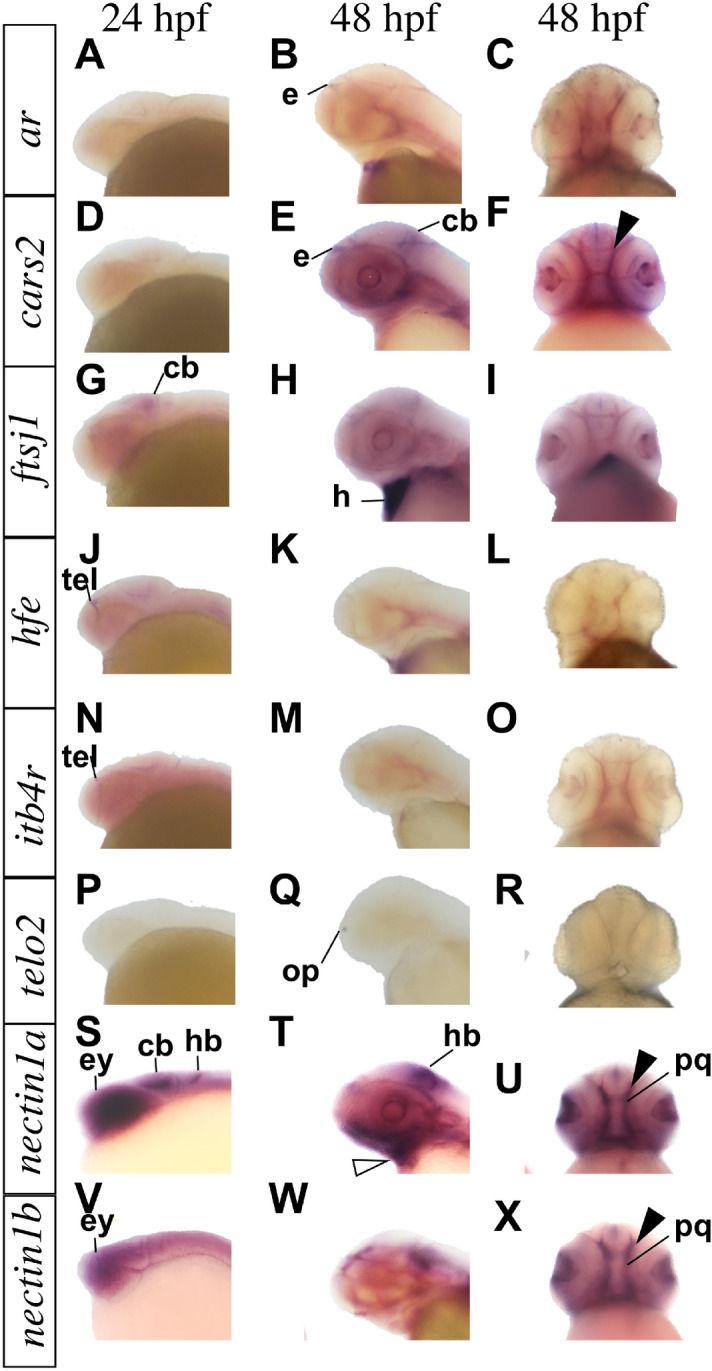
Whole-mount RNA in situ hybridization demonstrating genes expression in zebrafish. Genes expression pattern in lateral and ventral views at the indicated embryonic stages as hours per fertilization (hpf). *cars2, nectin1a* and *nectin1b* are expressed in zebrafish palate (solid arrow). *nectin1a* is expressed in the lower jaw at 48 hpf (hollow arrow). *cb* cerebellum, *e* epiphysis, *ey* eye, *h* heart, *hb* hindbrain, *op* olfactory placode, *pq* palate quadrate, *tel* telencephalon.

Zebrafish WISH was used to examine *ar, cars2, ftsj1, hfe, ltb4r, telo2, nectin1a* and *nectin1b* expression pattern in the craniofacial region across key developmental stages (Fig. 5). At 24 h post fertilization (hpf), *ftsj1* was expressed in the hindbrain, and *hfe* and *ltb4r* were expressed in the forebrain. We detected *nectin1a* and *nectin1b* transcripts in the eyes, diencephalon, midbrain and hindbrain at 24 hpf. At 48 hpf, *ar* expression was detected in the epiphysis*, cars2, nectin1a* and *nectin1b* were expressed in the palate (Fig. 5, solid arrow), and *nectin1a* was detected in the lower jaw (Fig. 5, hollow arrow).

**Figure 6:**
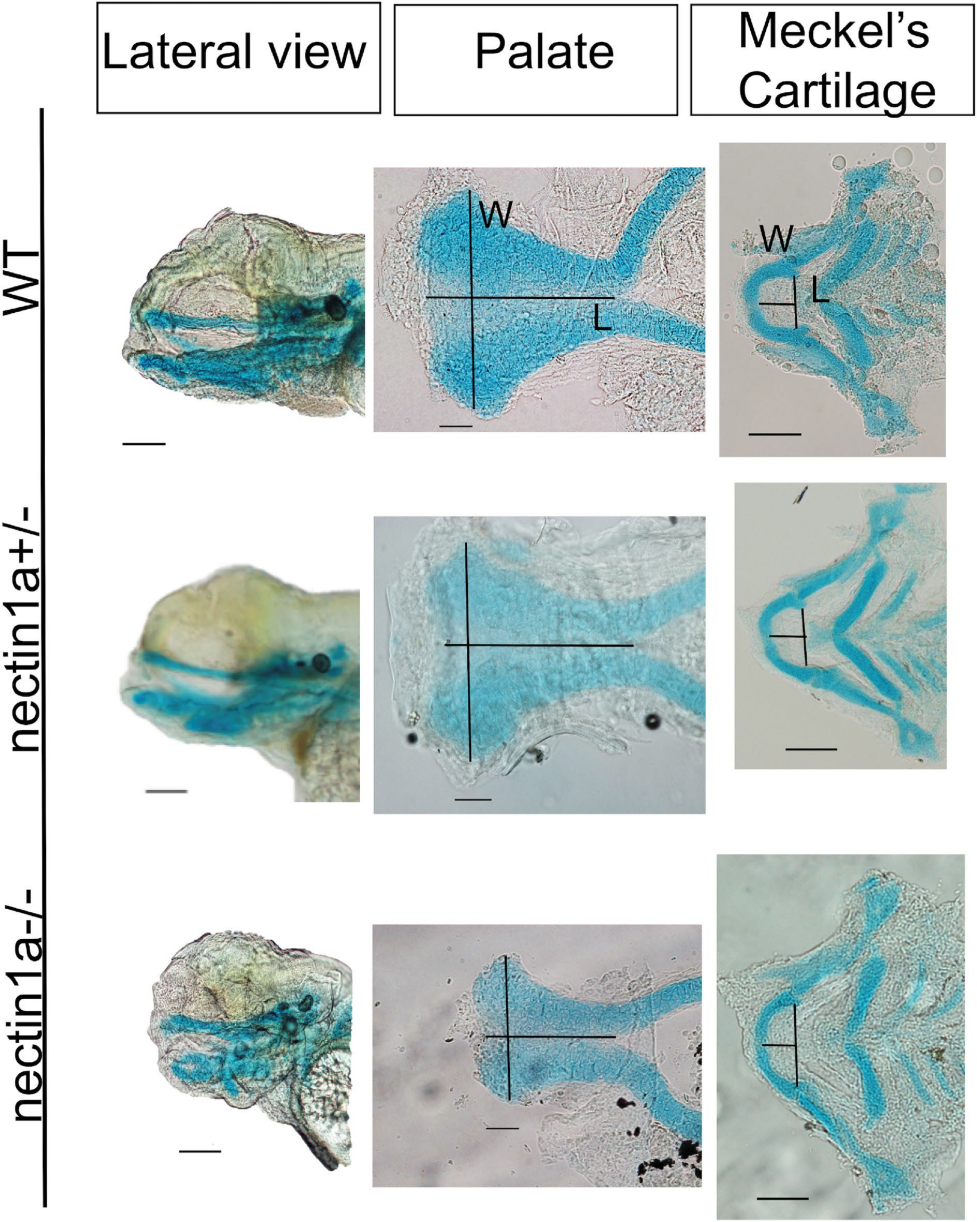
Alcian blue images for *nectin1a* zebrafish mutant compared to wild type at day 5. Top images: wild type alcian blue lateral view, palate and Meckel’s cartilage. Middle images: heterozygous *nectin1a* embryo alcian blue. Bottom images: homozygous *nectin1a* mutant lateral view. The length of the palate was measured from the anterior midpoint to the posterior midpoint of the palate. The width was measured as the maximum distance between the 2 lateral borders at the anterior area. The length of the Meckel’s cartilage was measured from the midline of the Meckel’s cartilage to the midline of an imaginary line drawn joining the joints between the Meckel’s cartilage and the palatoquadrate. The width was measured from the junction of the Meckel’s cartilage and the palatoquadrate of one side to the other side. Compared to wild type animals. *nectin1a* mutants have smaller and shorter palate, and shorter and wider Meckel’s cartilage. *L* length, *W* width. Scale bar: 10 μm.

To investigate if *nectin1a* is required for during normal craniofacial development, we analyzed the *nectin1a* mutant allele Ia021885Tg. Breeding of *nectin1a*+/− intercross generated embryos with Mendelian ratio (1 individual homozygous for the wild type allele: 2 heterozygous individuals with one wild type and one mutant allele: 1 individual homozygous for the mutant allele) demonstrating a mutant craniofacial phenotype, characterized by small head structures (Fig. 6). Using Alcain blue staining at 120 hpf, *nectin1a* mutants displayed dysmorphic craniofacial development with smaller and distorted palate and abnormal Meckel’s cartilage compared to age-matched wild type zebrafish embryos from the same intercross. These results show that *nectin1a* is genetically required for palate and mandible morphogenesis.

## Discussion

This study presented a discovery effort to identify low-frequency coding variants associated with normal-range human facial shape, by undertaking gene-based association tests on a carefully phenotyped human cohort followed by functional experiments of the association results. Overall, we demonstrated that part of the morphological variation of facial shape is attributable to low-frequency coding variants, and pinpointed putative functional genes involved. Seven genes (*AR*, *CARS2*, *FTSJ1*, *HFE*, *LTB4R*, *TELO2* and *NECTIN1*) were identified, with phenotypic effects in the area of cheek, chin, nose and mouth. Notably, *NECTIN1* is known to cause a syndrome characterized by facial dysmorphology. Using a zebrafish model, we confirmed the expression of *nectin1a* and *nectin1b* in the developing head and the abnormal craniofacial phenotype in *nectin1a* mutants, with the affected structures being highly consistent with the associated facial region in the human data analysis. Taken together, these findings support the contribution of low-frequency coding variants to the genetic architecture of normal-range facial shape.

The seven genes identified by the multivariate approach are for the first time implicated in normal facial morphology. Six of the seven genes (all but *cars2*) were expressed in embryonic craniofacial tissues in zebrafish, demonstrating their potential involvement in craniofacial development. Cellular processes/functions of these genes include metal ion transport (*HFE*), signaling (*AR*, *LTB4R*), tRNA metabolism (*CARS2, FTSJ1*), DNA repair (*TELO2*) and cell adhesion (*NECTIN1*). This diversity in their biological function led to a variety of enriched functional pathways/categories in the gene-set enrichment analysis, yet without a strong signal in any particular one, probably due to the small number of genes and the polygenic nature of facial morphology. With the exception of *NECTIN1*, the role of these genes in patterning craniofacial structures is unknown, and further investigation is needed to gain better understanding of how these genes may influence neural crest development during early morphogenesis and thus affect the face.

Previous GWASs and studies of facial dysmorphology have demonstrated that there are common genetic factors underlying normal-range facial variation and orofacial clefting^5,11,35^. Our findings suggest that low-frequency coding variants may also help explain this relationship. Although none of the other genes implicated here have been shown to be involved in craniofacial development, *NECTIN1* is an established player that has been linked to both syndromic and isolated forms of orofacial clefting^36–38^. Individuals with cleft lip/palate-ectodermal dysplasia syndrome (OMIM:225060) have distinctive facial features including an underdeveloped lower jaw^39^, which is consistent with the facial segment (chin) where the *NECTIN1* association was observed. Although not passing the genome-wide threshold, *NECTIN1* also yielded some signals in modules representing the nose and cheek (Fig. 3), additional facial regions affected in this syndrome. Different variants in *NECTIN1* are likely involved in normal-range variation and in craniofacial disorders, which may help explain apparent differences in phenotypic severity. Nectin-1 expression has been reported in migrating neural crest cells^40^, indicative of a possible role in cell movement and morphogenesis during craniofacial development. The Nectin-1 protein belongs to the subfamily of immunoglobulin-like adhesion molecules which are key components of cell adhesion junctions and play critical roles in the development of many tissues, including in the fusion of palatal shelves during palatogenesis^41^. A handful of *NECTIN1* mutations that can potentially disrupt gene function have been documented in non-syndromic cleft patients^42–44^. In the current study, two coding variants in *NECTIN1* contributed to the gene-level significance, both predicted to be deleterious. We performed lookups of the face-associated genes in a previous exome scan of a NSCL/P cohort^25^. *NECTIN1* yielded a small p-value of 0.004, although not passing the Bonferroni significance threshold. Two other genes, *TELO2* and *HFE*, did pass that threshold. These results are in line with previous evidence suggesting a role for same genes in normal and abnormal facial development.

Our zebrafish experiments provided a strong support for the relevance of *nectin1a* in palate and mandible development. The mutants displayed changes in the shape and size of both the palate and the Meckel’s cartilage, from which the mandibles evolved. This affected cartilage structure in zebrafish mutants aligns well with the associated human anatomical region (chin and mandible), where the effects of *NECTIN1* were observed in the MultiSKAT test. These findings for the first time demonstrate a role of *NECTIN1* in normal-range facial variation. We highlight the approach of interrogating human candidate genes in a biological context using the zebrafish model, where dynamic gene expression can be assayed in a high throughput fashion. Those candidate genes with spatiotemporal gene expression in the craniofacial domains then can be evaluated in functional studies, where mutants may already be available from large scale mutagenesis projects or can be generated by CRISPR mediated gene editing. We acknowledge that there are some important differences in the craniofacial anatomy between fish and mammals; thus, future verification in murine models would be warranted.

With the hierarchical facial segmentation, we were able to identify genetic effects at different scales. For example, the effects of *FTSJ1* were observed globally in the full face, and also locally in specific modules on the side of the face. By contrast, the effect of *NECTIN1* was confined to localized facial parts only. These patterns may help with understanding the mechanisms by which genes act along the growth of facial structure. Our multivariate data-driven phenotyping approach eliminates the need of preselecting traits, captures more variation in the facial shape, and is more effective for gene mapping.

The current study is an important extension and complement of our prior work on common SNPs^5^. Here we exclusively focused on coding variants with MAF below 1%, which have been omitted based on standard QC procedure from previous facial GWAS attempts. We compared results from this study to those from our prior GWAS^5^, and noted that common variants in or near (within 500 kb) the seven associated genes showed no evidence of association (p > 0.001 for all) with the same facial modules. This indicates that the current study generated distinct, non-overlapping knowledge on facial genetics, although it is possible that there are trans-acting common GWAS SNPs that regulate the expression of the seven identified genes during facial morphogenesis. Low-frequency variants showed larger magnitude of effects compared to common variants in our previous study^5^. It is necessary, however, to point out that this difference could partially or completely be a result of the drastically smaller groups of variant carriers, and we therefore refrain from overinterpreting the comparison.

Our study demonstrated the power of applying gene-based tests of low-frequency variants that are usually untestable individually. While some significant genes harbor variants with a small p-value in our single-variant association test, others would have been missed if not tested in aggregate. With a moderate sample size of 2329, it is highly desirable to collapse low-frequency variants into putative functional units and perform burden-style tests. In addition to an increase in power, another key benefit with analyzing low-frequency coding variants collectively is the improved biological interpretability compared to GWASs. The gene-centered design of coding variant tests facilities much clearer biological implications and options for experimental follow-up. Our success with the functional validation of *NECTIN1* provides a practical example. We expect future better-powered studies to discover more biological pathways emerging from analyses of low-frequency coding variants.

Replication of rare and low-frequency variant association signals presents unique challenges. The prominent barrier is the limited sample size. The low numbers or even absence of the carriers in independent populations hindered the replication efforts of our findings. Six out of the seven genes identified were not testable in a separate cohort of 664 participants due to a lack of variant carriers. Given our sample size and the ExomeChip design, this study was not adequately powered to identify genes harboring extra rare variants that may also contribute to facial traits. Although complex traits are not expected to have a large fraction of the heritability explained by rare and private variants, such variants may be influential, predictive, and actionable at the individual level. In this regard, whole exome or whole genome sequencing of large samples holds promise to give deeper insights into the role rare variants in facial morphology.

Like many other complex traits, research with a focus on uncovering the genetic architecture of facial morphology is confronted with the challenge of missing heritability^45,46^. Our study has extended the paradigm of genetic factors involved in facial morphology from common to low frequency variants and highlighted novel candidate genes that may lead to encouraging follow-ups. Given that rare and low-frequency genetic variation might be highly specific to certain populations, and facial shapes have distinctive ancestry features, future studies may benefit from extending the discovery of influential low-frequency variants to other ethnic groups.

## Supplementary Information

Supplementary Tables.

Supplementary Figures.

## Data Availability

All of the genotypic markers are available to the research community through the dbGaP controlled-access repository (https://dbgap.ncbi.nlm.nih.gov/) at accession phs000949.v1.p1. The raw source data for the phenotypes—the 3D facial surface models—are available for the 3D Facial Norms dataset through the FaceBase Consortium (http://www.facebase.org). Access to these 3D facial surface models requires proper institutional ethics approval and approval from the FaceBase data access committee. KU Leuven provides the spatially dense facial mapping software, free to use for academic purposes: MeshMonk (https://github.com/TheWebMonks/meshmonk).
