# Supplementary material for: Impact of low-frequency coding variants on human facial shape
Source: Sci Rep. 2021 Jan 12;11:748. doi: 10.1038/s41598-020-80661-y (PMC7804299; doi:10.1038/s41598-020-80661-y)

Impact of low-frequency coding variants on human facial shape

Dongjing Liu[1], Nora Alhazmi[2,3], Harold Matthews[4,5], Myoung Keun Lee[6], Jiarui Li[7], Jacqueline T. Hecht[8], George L. Wehby[9], Lina M. Moreno[10], Carrie L. Heike[11], Jasmien Roosenboom[6], Eleanor Feingold[1,12], Mary L. Marazita[1,6], Peter Claes[4,7], Eric C. Liao[13], Seth M. Weinberg[1,6*], John R. Shaffer[1,6*]

Supplementary Figures

Fig S1. Implicated genes across broad facial regions from recent GWAS of common genetic variants (White et al. 2020 [1])

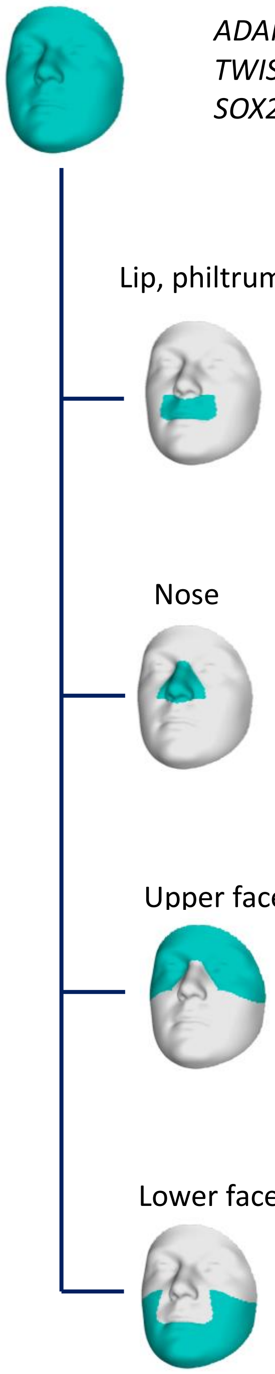

Full face

ADAM15, NT5C1B, PKDCC, HOXD1, COL8A1, SLCO2A1, BMP5, TWIST1, DPYSL2, PXDNL, VPS13B, EMX2, ALX4, PTHLH, ALX1, TGDS-SOX21, PAX9, BCL11B, DNMT3B, EDA

Lip, philtrum

PRRX1, SIX3, CALCRL, SATB2, CD96, GATA2, MSX1, PRDM5, MCC, PRDM1, HOXA2, MSRA, CCDC26, FREM1, C10orf11, FGFR2, KCNQ1, NAV3, ALX1, SIX1, RAD51B, RPGRIP1L, FOXC2, NOG, CYP24A1

Nose

PAX7, CAPZB, MATN1, c1orf87, LPHN2, OSR1, PKDCC, SIX2, NCAPH, PAX3, SRGAP3-SETD5, FOXP1, EPHB3, FGFRL1, LEF1, FABP6, DAAM2, SUPT3H, RUNX2, RCAN2, TFAP2B, PKHD1, GLI3, IGFBP3, HGF, FEZF1, SFRP1, TRHR, PTCH1, GDF10, ARID5B, TCTN3, FGF8, TBX3, SPRY2, TGDS-SOX21, IRS2, BMP4, ADAMTSL3, SOX9, SETBP1, TCF4, TSHZ1, KCTD15, MKKS, PAX1, FOXA2, BMP7

Upper face

PRDM16, PLPP3, SLC44A5, WARS2, OSR1, THADA, EFEMP1, SPRED2, MEIS1, DYSF, INSIG2, EN1, ZEB2, THRB, ARL13B, PITX2, MYOZ2, FGF10, FGF18, MYLK4, COL11A2, TBX18, EYA4, MTFR2, COL28A1, SEMA3A, DGK1, SNAI2, PKIA, POP1, DMRT2, ELP1, LMX1B, EMX2, SOX6, ALX4, A2ML1, LRIG3, HMGA2, PCCA, BCL11B, MEIS2, ITGA11, NOG, BMP2, PAX1, FBLN1, NLGN4X, FAM133A, ZIC3

Lower face

TBX15, WARS2, PRRX1, CRB1, MARK1, OSR1, BCL11A, PNO1, FBLN7, MTX2, SATB2, EPHB3, STX18, FAT4, HAND2, HPGD, FBN, RSPO3, DLX6, WNT16, TRPS1, NAV3, DACT1, GREM1, THSD4, DPH1, SHBG, TBX4

[1] White,J.D., Indencleef,K., Naqvi,S., Eller,R.J., Hoskens,H., Roosenboom,J., Lee,M.K., Li,J., Mohammed,J., Richmond,S., *et al.* (2020) Insights into the genetic architecture of the human face. *Nat Genet*, **66**, 101–9.

Fig S2. Q-Q plot of gene-based MultiSKAT tests by facial module. Genomic inflation factor λ is shown at the top left corner of each subfigure. There was little evidence for systematic inflation in p-values.

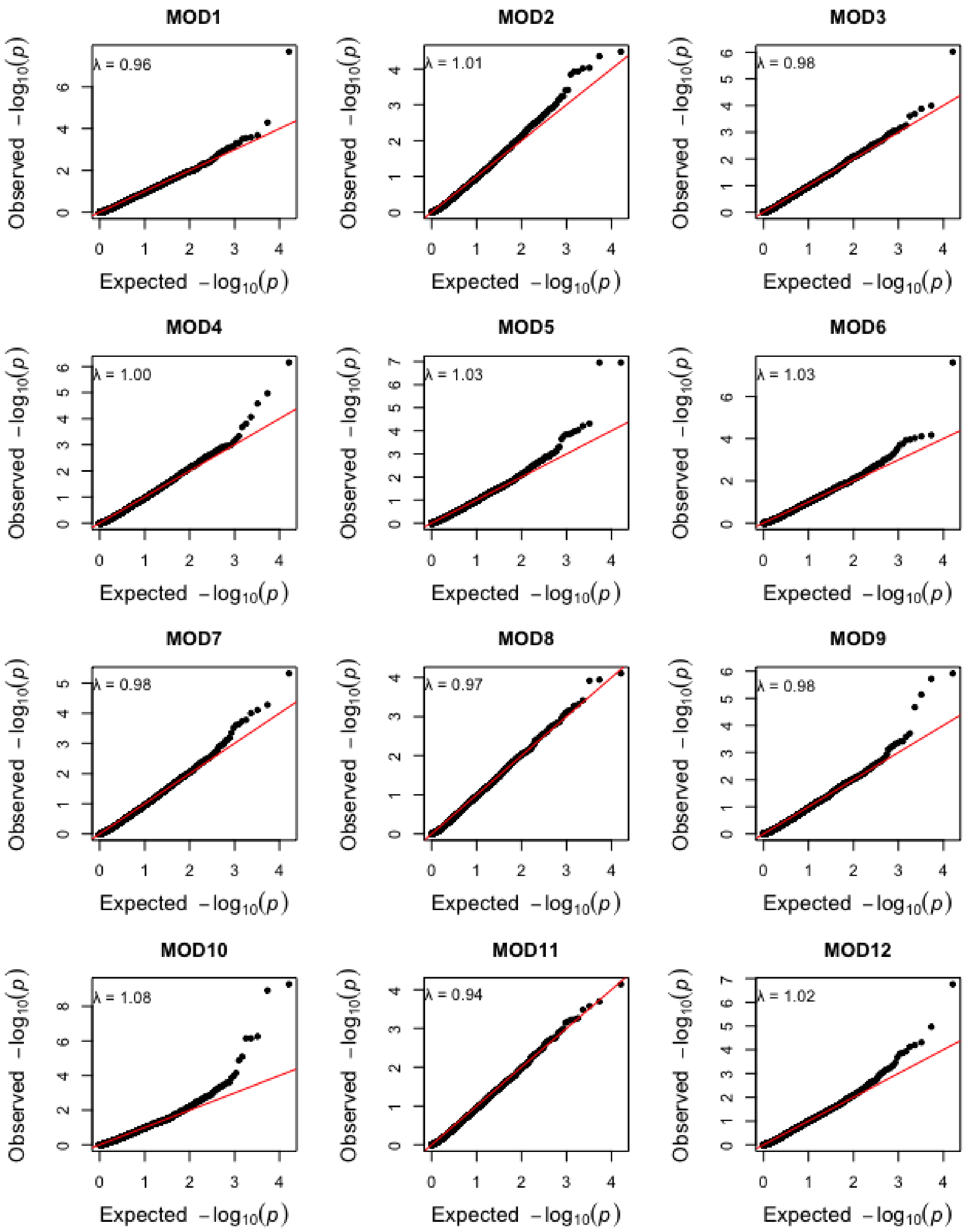

Fig S2. Q-Q plot of gene-based MultiSKAT tests by facial module (Cont)

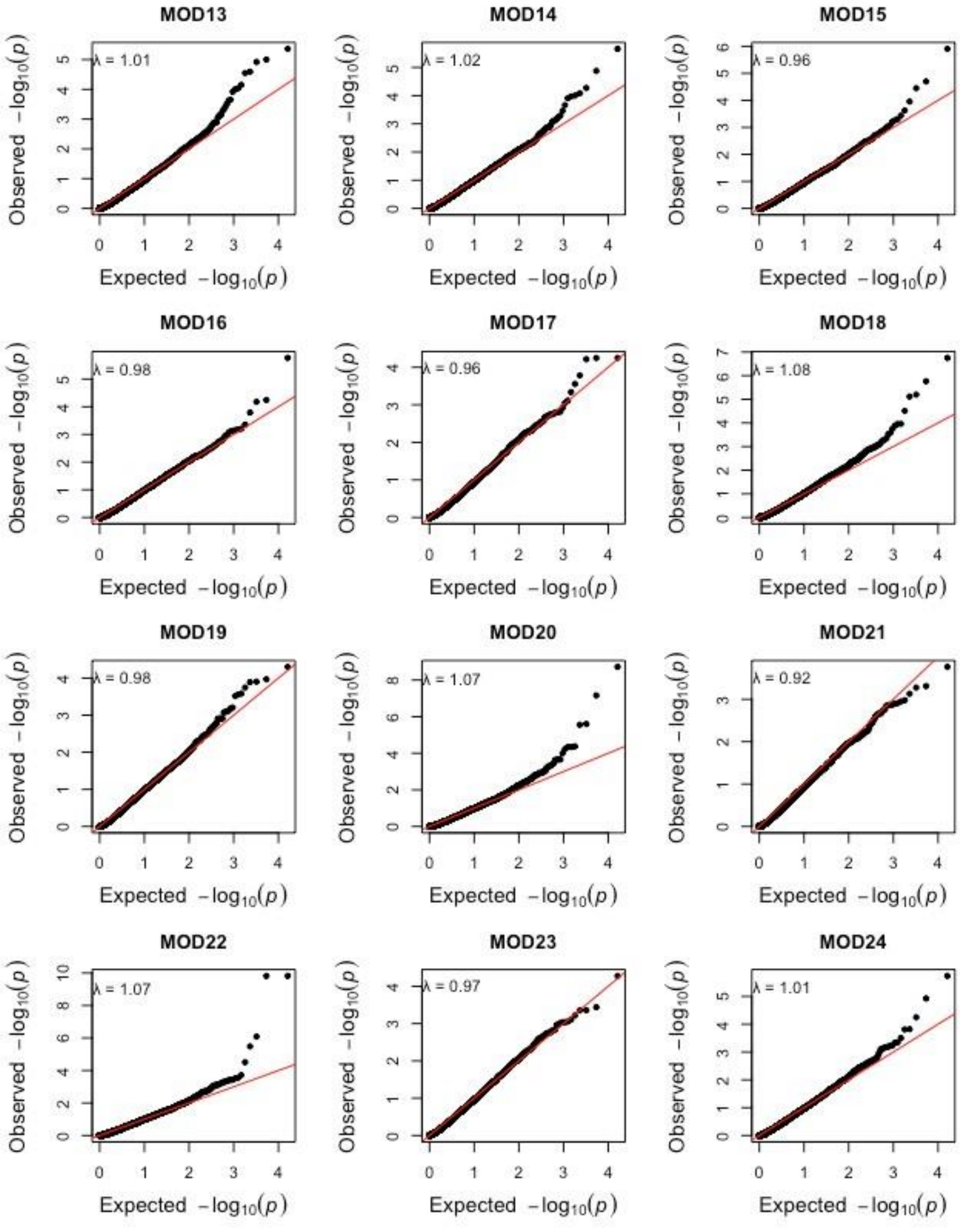

Fig S2. Q-Q plot of gene-based MultiSKAT tests by facial module (Cont)

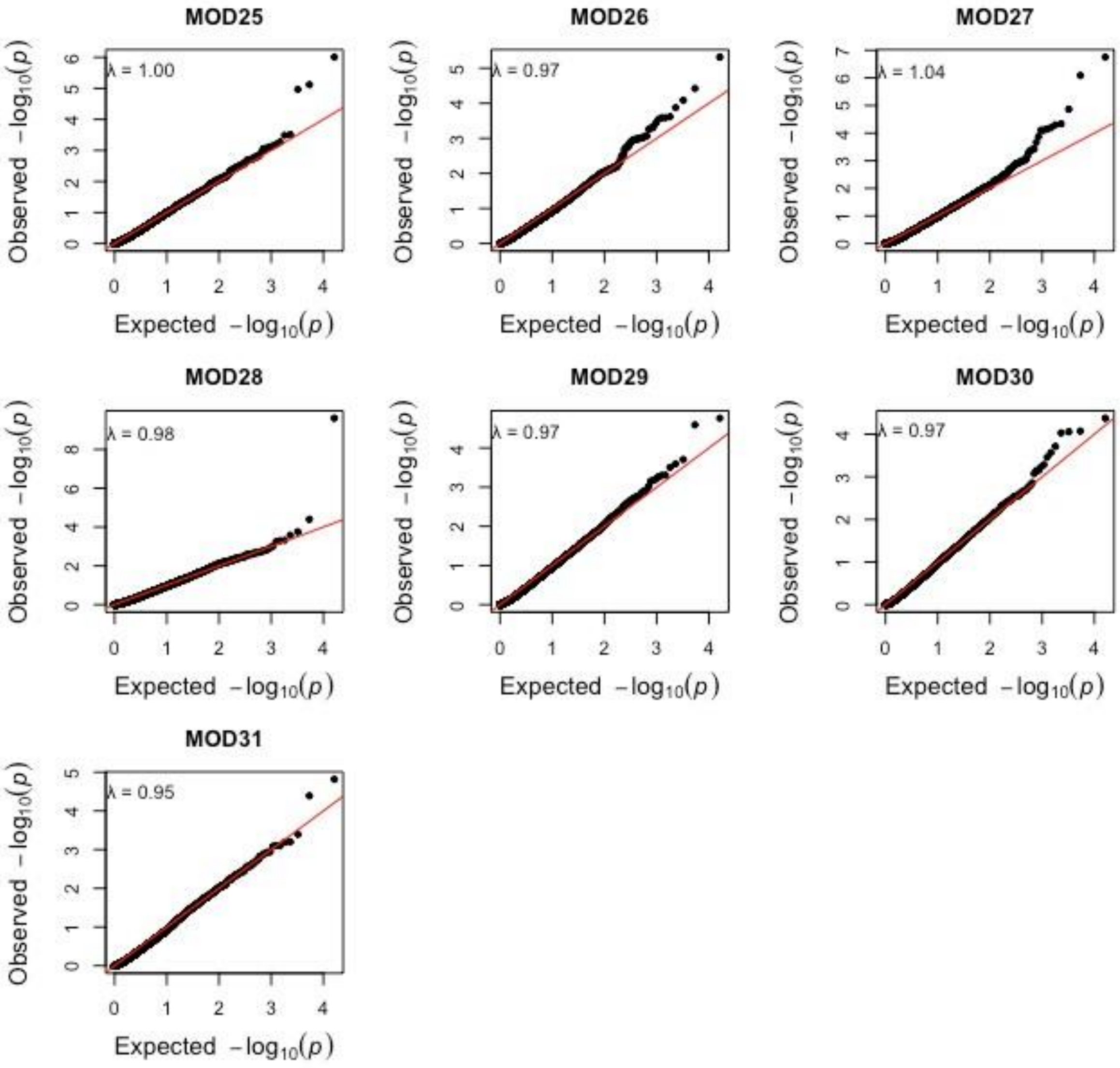

Fig S3. FUMA enrichment results

a) GO biological process

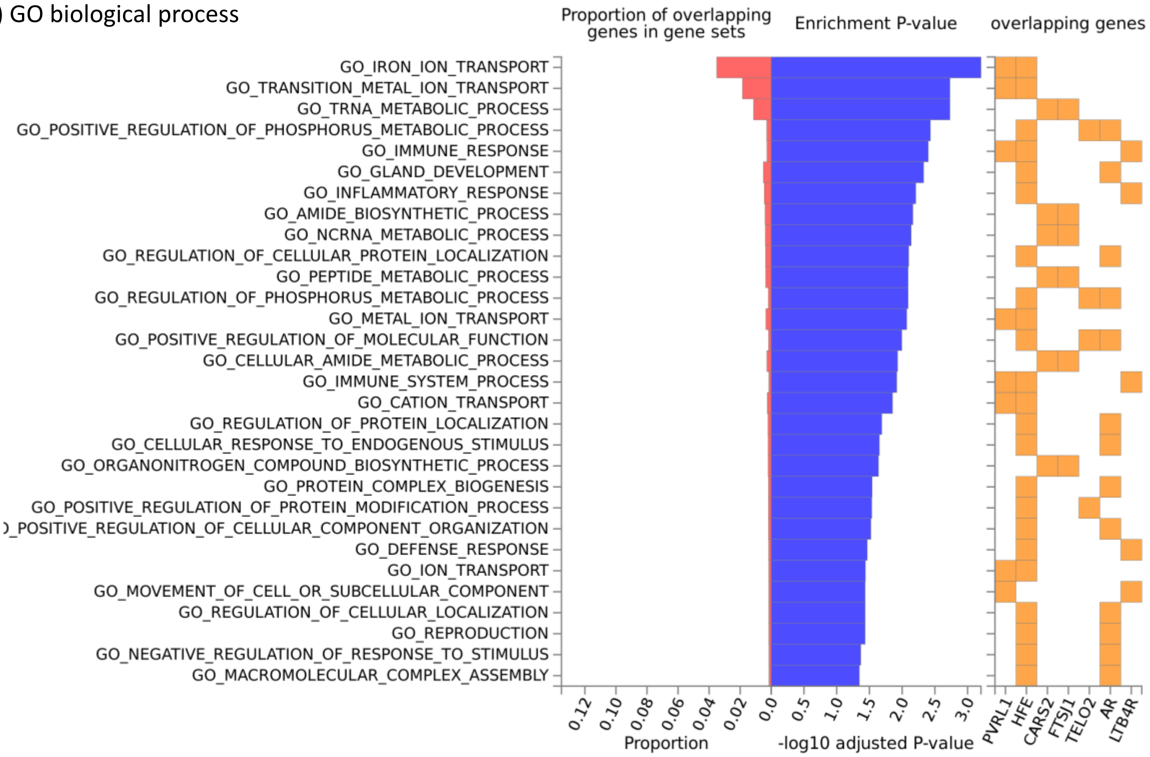

b) GO molecular function

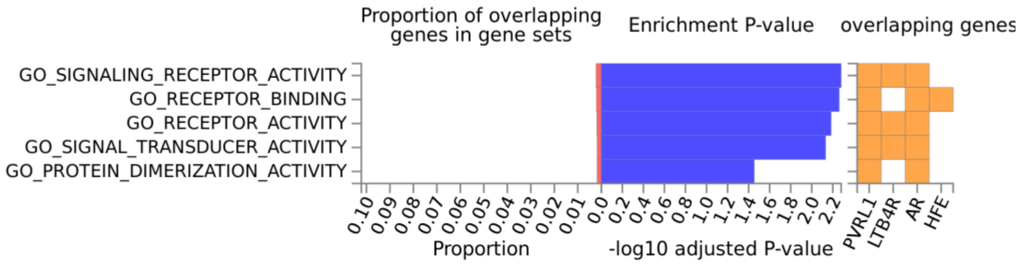

c) GWAS catalog

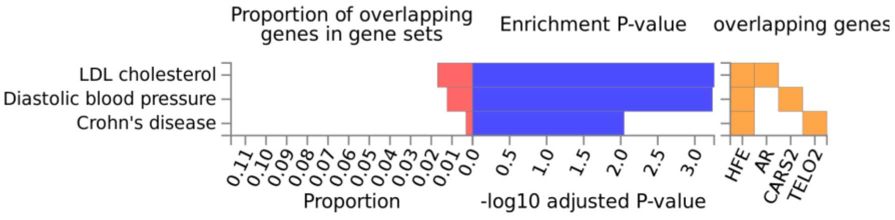

Fig S4. GTEx expression of MultiSKAT significant genes in tissues relevant to facial morphology. Dendrogram denotes similarity in expression level. TPM, transcripts per million

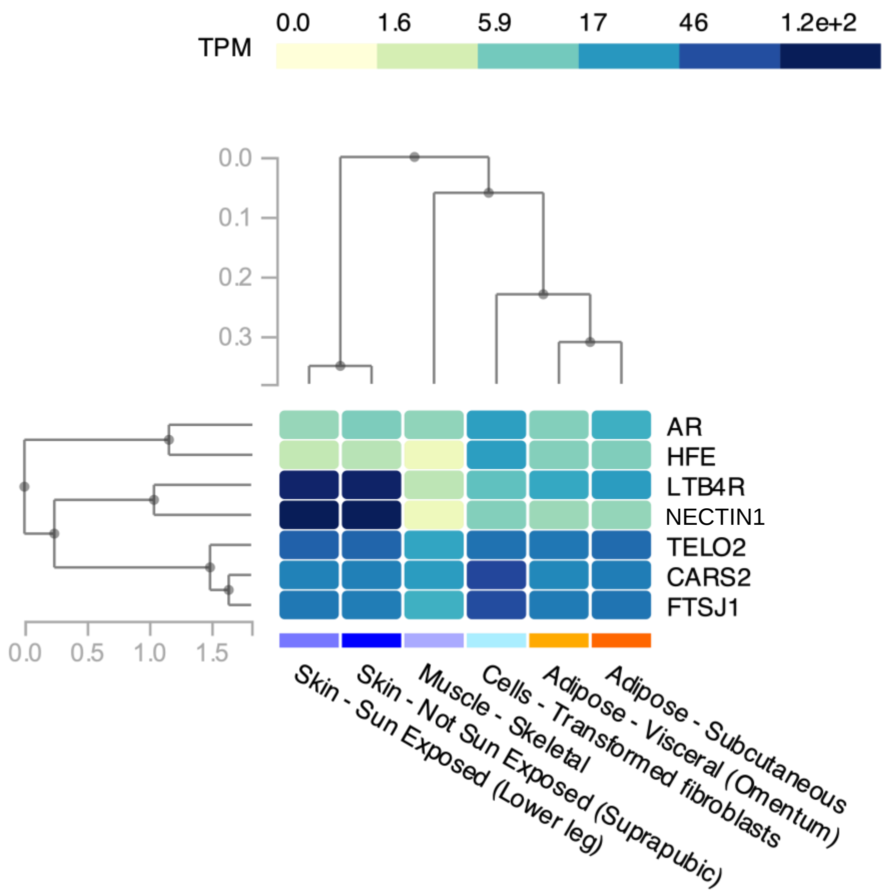

Fig S5. Magnitude of variant effect on facial modules, quantified by the Euclidean distance between averaged faces of different genotype groups. The 95% confidence interval was obtained by 5000 bootstraps. The farther away the blue (common) or red (low-freq) rectangular boxes fall from line x=0, the larger the group distances and the greater the magnitude of effects. Common variants that yielded significant GWAS association in the same cohort with the same modules are used as a comparison to low-frequency variants. Genotype groups column indicates the two groups of people of whom the faces were averaged and distance was computed. For example, 0 vs 1/2 means major allele homozygotes vs the remaining. The following two columns indicate sizes of the two groups in comparison. Low-frequency variants had large effects compared to previously reported common variants, although this could be a result from the much smaller size of carrier group and may not reflect genuine greater effects of low-frequency variants.

Module	Variant	Genotype groups	N_noncarriers	N_carriers
Common variants				
10	rs227833	0 vs 1	1266	909
		0 vs 2	1266	153
	rs34472363	0 vs 1	843	1130
		0 vs 2	843	329
	rs5821892	0 vs 1	750	1150
		0 vs 2	750	414
20	rs34472363	0 vs 1	843	1130
		0 vs 2	843	329
22	rs2058084	0 vs 1	891	1116
		0 vs 2	891	316
27	rs10238953	0 vs 1	1720	565
		0 vs 2	1720	44
	rs2759661	0 vs 1	1452	776
		0 vs 2	1452	101
Low-freq variants				
10	rs151097801	0 vs 1/2	2325	4
	rs117788141	0 vs 1/2	2325	4
	rs140903666	0 vs 1/2	2319	10
	rs144863771	0 vs 1/2	2319	10
	rs147858841	0 vs 1/2	2324	5
18	rs142280455	0 vs 1/2	2324	5
	rs137852591	0 vs 1/2	2324	5
20	rs143666989	0 vs 1/2	2324	5
	rs148153989	0 vs 1/2	2325	4
22	rs149342416	0 vs 1/2	2323	6
	rs143662783	0 vs 1/2	2325	4
27	rs142863092	0 vs 1/2	2325	4
	rs137991779	0 vs 1/2	2324	5
28	rs142932029	0 vs 1/2	2326	3
	rs201095751	0 vs 1/2	2325	4

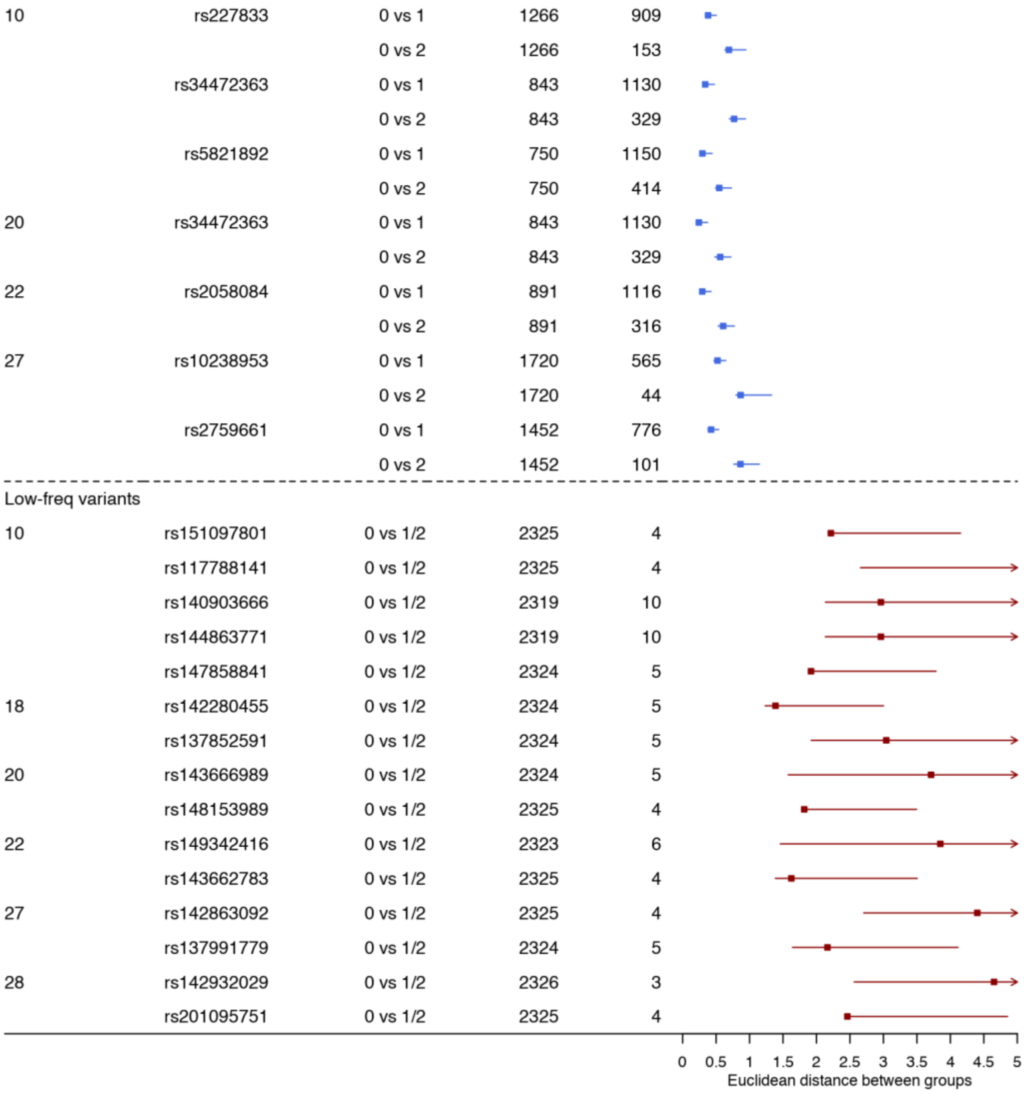

Supplement: Supplementary file 2 — Supplementary Figures. [file 41598_2020_80661_MOESM2_ESM.pdf]
